# SIRT1 Silences L1 Retrotransposons by Stabilizing Heterochromatin‐Modifying Complexes

**DOI:** 10.1111/acel.70689

**Published:** 2026-08-30

**Authors:** Xiaona Wang, Tianbo Li, Huanyin Tang, Meng Xiao Liu, Qinghua Peng, Xiao Huang, Zhiyong Mao, Ying Jiang, Anke Geng

**Affiliations:** ^1^ Shanghai Key Laboratory of Maternal Fetal Medicine, Clinical and Translational Research Center of Shanghai First Maternity and Infant Hospital, Frontier Science Center for Stem Cell Research, School of Life Sciences and Technology Tongji University Shanghai China

**Keywords:** cellular senescence, genomic stability, L1, SIRT1

## Abstract

SIRT1, a sirtuin family member, has been extensively documented to be closely linked to aging and aging‐related disease. Cellular senescence is a state of irreversible cell cycle arrest that functions as a key driver of aging. During cellular senescence, LINE‐1 (L1) retrotransposable elements become transcriptionally activated and stimulate a type‐I interferon (IFN‐I) response. L1 activity has been strongly linked to aging and a variety of age‐related disorders. However, whether SIRT1 influences cellular senescence through the transposable element L1 remains unknown. In this study, we discovered that SIRT1 significantly suppresses L1 retrotransposition. Under quiescent conditions, SIRT1 exhibits increased enrichment at the L1 5′‐UTR region. This recruitment enhances its interaction with the heterochromatin‐regulatory factors Lamin B1 and KAP1, subsequently elevating H3K9me3 levels. This repressive chromatin mark inhibits L1 transcription, thereby maintaining genomic stability and delaying cellular senescence. Consistent with these observations, SIRT1‐deficient cell lines exhibited a marked reduction of the interaction of Lamin B1 and KAP1 and a reduced presence of these factors at the L1 5′‐UTR. Consequently, the elevated L1 transcription resulting from SIRT1 deficiency activated the cGAS‐STING pathway and ultimately triggered cellular senescence, an effect that was rescued by treatment with 3TC (a nucleoside reverse transcriptase inhibitor). In summary, our findings demonstrate that SIRT1 suppresses L1 retrotransposition by recruiting heterochromatin factors, thereby delaying cellular senescence and providing new insights with implications for delaying aging and mitigating age‐related pathologies.

## Graphical abstract

SIRT1 binds the LINE‐1 5′‐UTR and recruits the heterochromatin modifiers Lamin B1 and KAP1 to maintain H3K9me3 levels, thereby suppressing LINE‐1 retrotransposition. SIRT1 deficiency enhances LINE‐1 transcription, leading to upregulation of senescence‐associated secretory phenotype factors, activation of the cGAS‐STING pathway, and consequent cellular senescence.

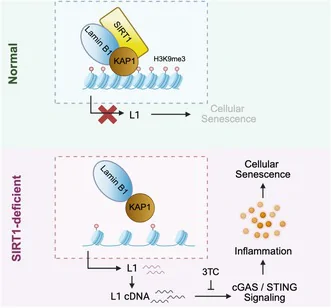

## 1 Introduction

Cellular senescence is a key driver of aging and age‐related pathologies, with studies demonstrating the progressive accumulation of these cells in some tissues of aging mice and primates (Herbig et al. 2006; Jeyapalan et al. 2007; Wang et al. 2009). Genetic or pharmacological clearance of senescent cells has been demonstrated to extend both healthspan and longevity in naturally aged mice (Xu et al. 2018). Furthermore, cellular senescence is implicated in a range of pathologies, including hepatic steatosis, obesity‐associated metabolic syndrome, Type I and II diabetes, atherosclerosis, as well as Alzheimer's and Parkinson's diseases (Biga et al. 2025). Therefore, elucidating the molecular mechanisms underlying cellular senescence may offer valuable insights into strategies for mitigating age‐related diseases or potentially reversing aspects of cellular aging.

Long interspersed elements‐1 (LINE‐1, L1) belongs to the non‐LTR retrotransposon family and is the only active autonomous transposable element in humans. It comprises approximately 17% of the human genome (Beck et al. 2010; Cervantes‐Ayalc et al. 2020). The L1‐encoded proteins (ORF1p and ORF2p) can mobilize nonautonomous retrotransposons, a process generating nearly a third of our genome (Cashen et al. 2024; Beck et al. 2011; Baldwin et al. 2024). L1 activity can significantly impact genomic integrity and has been strongly linked to aging and aging‐associated diseases such as cancer. Consistently, elevated L1 activity is observed in senescent and cancer cells (De Cecco et al. 2013; Rodic and Burns 2013). Several studies indicate that aging is associated with a reduction in heterochromatin or its key regulatory factors, resulting in increased L1 expression (Lee et al. 2020; Li et al. 2024; Tsurumi and Li 2012). Histone H3 lysine 9 trimethylation (H3K9me3) is a characteristic epigenetic mark of constitutive heterochromatin. Methyltransferases SUV39H1/2‐mediated H3K9me3 deposition plays a specific role in repressing intact LINE elements in the embryonic stem cells epigenome (Bulut‐Karslioglu et al. 2014; Guerra et al. 2021). For more ancient L1s, with plausibly limited transcriptional activity, H3K9me3 accumulation is mediated by the KRAB/KAP1 pathway (Castro‐Diaz et al. 2014).

Sirtuins, an evolutionarily conserved family of NAD^+^‐dependent deacetylases and the mammalian homologs of the yeast longevity protein Sir2, comprise seven members (SIRT1–SIRT7) in mammals (Almeida and Porter 2019; Haigis and Sinclair 2010). Sirtuin family members participate in regulating numerous cellular processes including metabolism, mitochondrial homeostasis, autophagy, DNA repair, apoptosis, redox balance, and aging (Houtkooper et al. 2012; Alves‐Fernandes and Jasiulionis 2019; Geng et al. 2024; Chen et al. 2019). SIRT1, an important member of the sirtuin family, has been found to participate in the regulation of cellular senescence and organismal aging by deacetylating histones and non‐histone factors. For example, SIRT1 reduced the H3K9/14ac level in the IL‐11 promoter region, inhibited the TGF‐β1/IL‐11/MEK/ERK signaling pathway, and prevented senescence‐associated pulmonary fibrosis (Zhou et al. 2022). At the same time, SIRT1 with its partner Nkx2‐1 regulated the expression of Ox2r to prolong the lifespan of mice (Satoh et al. 2013). In addition, SIRT1 protein levels gradually decrease in senescence and aging (Xu et al. 2020). Previous studies have demonstrated that SIRT6 and SIRT7, also the members of sirtuin family, can inhibit the L1 retrotransposon, thereby inhibiting cellular senescence. This role is further supported by in vivo evidence, as mice lacking SIRT6 have significantly shortened lifespan and healthspan (Kawai and Montgomery 1987; Simon et al. 2019; Bi et al. 2020; Van Meter et al. 2014; Mostoslavsky et al. 2006). However, whether SIRT1 can regulate the L1 genome to directly affect cellular senescence has not yet been studied.

In our study, we find that SIRT1 represses L1 retrotransposition independent of its deacetylase activity. SIRT1 deficiency exacerbated ORF2‐induced genomic instability and promotes stress‐induced cellular senescence, increases SASP expression, and activates the cGAS‐STING pathway, which is rescued by using the nucleoside reverse transcriptase inhibitor lamivudine (also known as 3TC). Mechanistically, SIRT1 binds to the L1 5′‐UTR and recruits heterochromatin modifiers Lamin B1 and KAP1 to regulate H3K9me3 levels, thereby repressing L1 retrotransposition. Our work elucidates a novel function of SIRT1 in stress‐induced cellular senescence, which holds significant implications for delaying the aging process and mitigating age‐related diseases.

## 2 Results

### 2.1 SIRT1 Inhibits L1 Retrotransposition by Suppressing 5′‐UTR Activity

**FIGURE 1:**
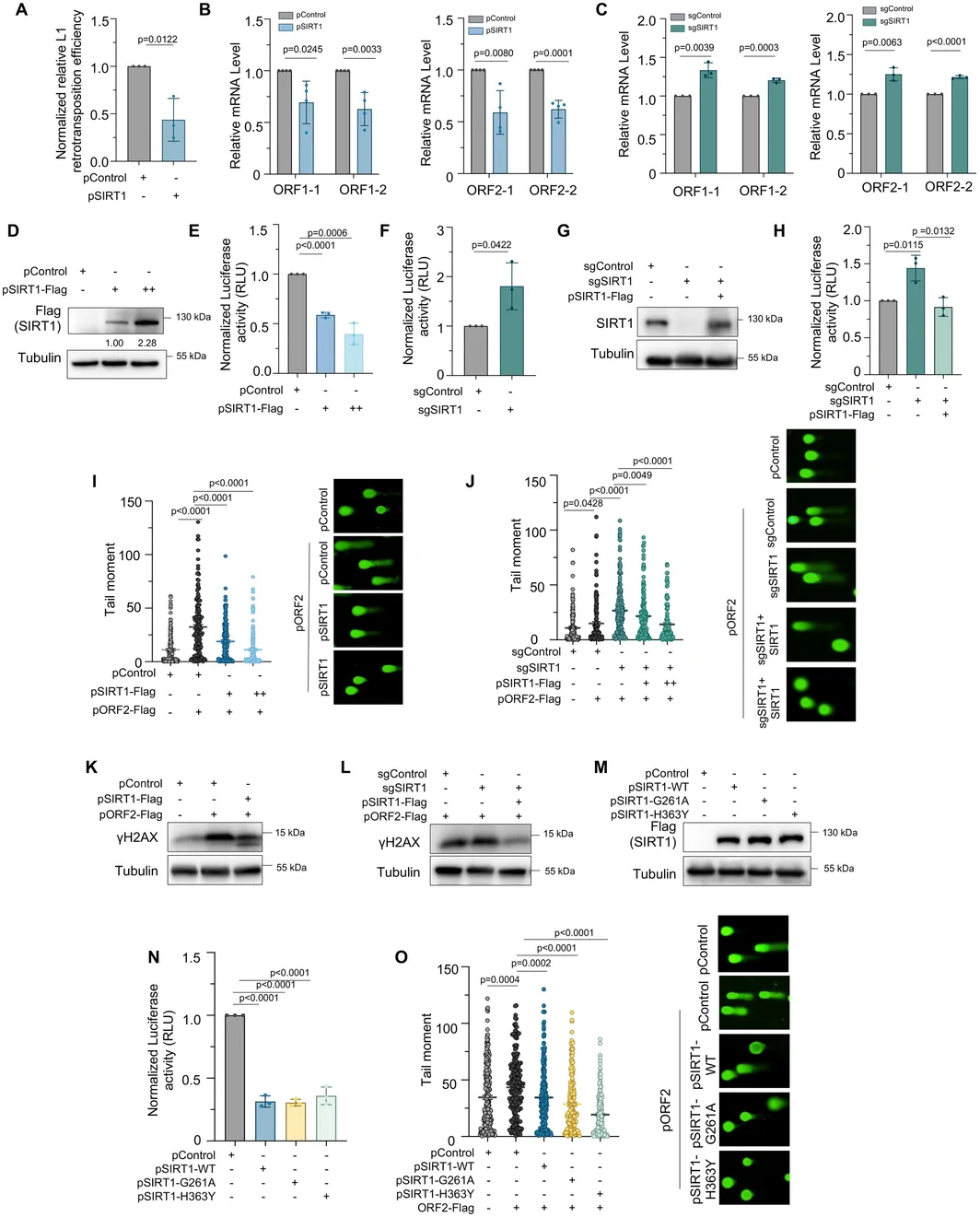
SIRT1 inhibits L1 retrotransposition by suppressing 5′‐UTR activity. (A) Effects of SIRT1 overexpression on L1 retrotransposition efficiency in HeLa cells. The HeLa cells were transfected with the control vector or the SIRT1 overexpression vector. (B) Analysis of L1 mRNA levels in HeLa cells transfected with the control vector or the SIRT1 overexpression vector. (C) Analysis of L1 mRNA levels in control and SIRT1‐deficient HeLa cells. (D) Western blot analysis of SIRT1 expression in HeLa cells transfected with the control vector or the SIRT1 overexpression vector. (E) Analysis of the firefly luciferase gene driven by 5′‐UTR in HeLa cells. The HeLa cells were transfected with the control vector or the SIRT1 overexpression vector. (F) Analysis of the firefly luciferase gene driven by 5′‐UTR in control and SIRT1‐deficient HeLa cells. (G) Western blot analysis of restoring SIRT1 in control and SIRT1‐deficient cells. (H) Restoring SIRT1 in SIRT1‐deficient cells abolished the stimulatory effect of knocking out SIRT1 on L1 5′‐UTR activity. (I) The genomic instability analysis in HeLa cells using the alkaline comet assay. The HeLa cells were transfected with the control vector or the SIRT1 overexpression vector, and ORF2 overexpressed in the last three groups. The tail moment was employed as the measure of genomic instability, and at least 100 cells were analyzed using the software CometScore. Representative images of the comet assay. (J) Restoring SIRT1 in SIRT1‐deficient cells abolished the stimulatory effect of knocking out SIRT1 on genomic instability. Representative images of the comet assay. (K) Western blot analysis of γH2AX in HeLa cells. The HeLa cells were transfected with the control vector or the SIRT1 overexpression vector, and ORF2 overexpressed in the last two groups. (L) Western blot analysis of γH2AX in control and SIRT1‐deficient HeLa cells, rescued with SIRT1 overexpression vector, and ORF2 overexpressed in each group. (M) Western blot analysis of SIRT1 and its enzyme activity mutants in HeLa cells. (N) Analysis of the firefly luciferase gene driven by 5′‐UTR in HeLa cells. The HeLa cells were transfected with the control vector or the SIRT1 and its enzyme activity mutant overexpression vector. (O) The genomic instability analysis in HeLa cells using the alkaline comet assay. The HeLa cells were transfected with the control vector or the SIRT1 and its enzyme activity mutant overexpression vector, and ORF2 overexpressed in the last four groups. The tail moment was employed as the measure of genomic instability, and at least 100 cells were analyzed using the software CometScore. Representative images of the comet assay. A, B, C, E, F, H, N: bars represent mean ± SD of biological replicates. Two‐tailed Student's *t*‐test. I, J, O: Bars represent mean ± SEM Mann–Whitney *U* test.

To investigate the role of SIRT1 in regulating L1 retrotransposition, we employed a well‐characterized GFP‐based reporter assay to quantify L1 retrotransposition efficiency (Figure S1A) in HeLa cells (Ostertag et al. 2000). Our results demonstrated that SIRT1 overexpression significantly suppressed L1 retrotransposition (Figure 1A, Figure S1B,C). Conversely, siRNA‐mediated knockdown of SIRT1 resulted in an increase in L1 retrotransposition efficiency (Figure S1D–F).

We next examined whether SIRT1 modulates L1 at the transcriptional level. L1 mRNA levels were reduced ~30% in SIRT1 overexpressing cells (Figure 1B). We established a CRISPR/Cas9‐mediated SIRT1 knockout cell line, and the results demonstrated that the absence of SIRT1 led to an increase in L1 transcription (Figure 1C, Figure S1G). Furthermore, depletion of SIRT1 also resulted in elevated L1 mRNA levels in IMR90 primary cells (Figure S1H,I). Consistent with this, SIRT1‐knockdown MEFs exhibited similarly increased L1 mRNA levels relative to control MEFs (Figure S1J,K). Since L1 transcription is initiated by an internal promoter within its 5′‐UTR, we assessed the impact of SIRT1 on L1 promoter activity using a luciferase reporter plasmid driven by the L1 5′‐UTR (Van Meter et al. 2014). SIRT1 was found to inhibit L1 5′‐UTR activity in a dose‐dependent manner (Figure 1D,E). Furthermore, SIRT1 deficiency enhanced 5′‐UTR activity by approximately 2 fold (Figure 1F), and this effect was reversed upon reintroduction of SIRT1 (Figure 1G,H). Similarly, we found that overexpressing SIRT1 inhibited L1 5′‐UTR activity, while depleting SIRT1 enhanced 5′‐UTR activity in IMR90‐SVLT fibroblasts (Figure S1L,M).

Given previous reports that L1 ORF2 protein induces genomic instability (Zhen et al. 2023), we evaluated the effect of SIRT1 on 5′‐UTR promoter driven ORF2‐mediated DNA damage using the alkaline comet assay. The alkaline comet assay is a technically simple and sensitive method for detecting DNA damage at the single‐cell level, including strand breaks and other lesions that are converted into strand breaks under alkaline conditions. The tail moment is a descriptor used to calculate the overall extent of DNA damage (Møller et al. 2020). Overexpression of SIRT1 reduced the tail moment in a dose‐dependent manner (Figure 1I). In contrast, SIRT1 deficiency exacerbated ORF2‐induced genomic instability, and reintroduction with wild‐type SIRT1 rescued this phenotype (Figure 1J, Figure S1N). These phenotypes can also be confirmed in IMR90‐SVLT fibroblasts (Figure S1O,P).

Consistent with these findings, SIRT1 overexpression decreased ORF2‐induced γH2AX accumulation (Figure 1K), while SIRT1 knockdown enhanced it (Figure S1Q). Re‐expression of SIRT1 in knockout cells rescued γH2AX levels (Figure 1L).

To determine whether the enzymatic activity of SIRT1 is required for its suppressive function, we generated two catalytically inactive mutants, G261A and H363Y (Nemoto et al. 2005; Pfister et al. 2008) (Figure 1M). Consistently, results indicated that SIRT1 inhibits L1 5′‐UTR activity and genomic instability in an enzyme activity‐independent manner (Figure 1N,O).

### 2.2 SIRT1 Deficiency Drives Cellular Senescence and Is Rescued by 3TC Treatment

**FIGURE 2:**
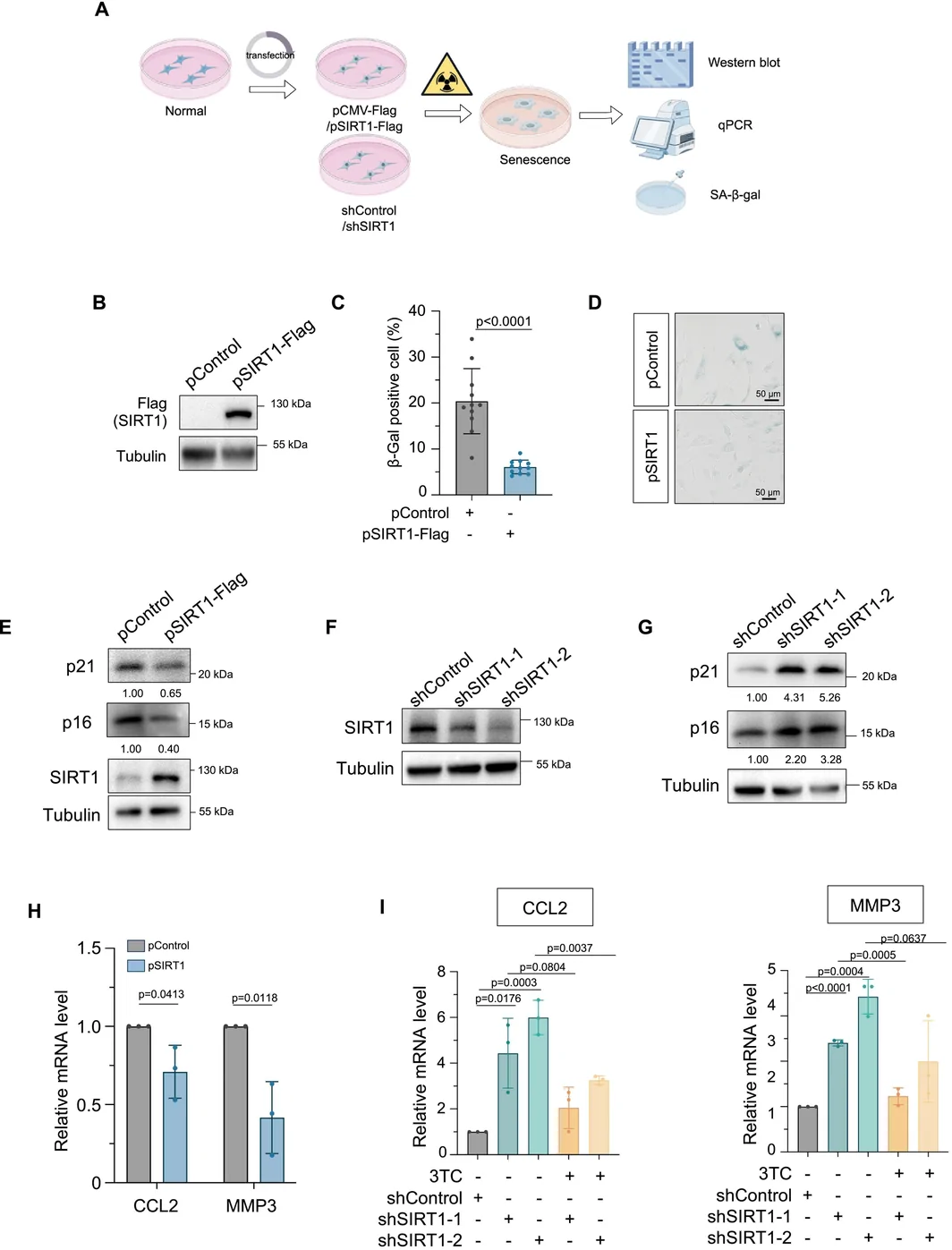
SIRT1 suppresses cellular senescence and this effect is rescued by 3TC treatment. (A) Schematic of induction in control, SIRT1‐overexpressing and SIRT1 knockdown cells post‐IR. (B) Western blot analysis of SIRT1 expression in HCA2‐hTERT cells. The HCA2‐hTERT cells were transfected with the control vector or the SIRT1 overexpression vector. (C) Quantification of SA‐β‐gal positive HCA2‐hTERT cells. HCA2‐hTERT cells were exposed to 10 Gy of X‐ray irradiation and transfected with the control vector or the SIRT1 overexpression vector for 7 days. Images from different areas of the plate were taken and quantified. (D) Representative images of SA‐β‐gal staining in HCA2‐hTERT cells, which were transfected with the control vector or the SIRT1 overexpression vector (Scale bar: 50 μm). (E) Western blot analysis of p21 and p16 in control and SIRT1‐overexpressing HCA2‐hTERT cells. (F) Western blot analysis of SIRT1 expression in control and SIRT1‐knockdown HCA2‐hTERT cells. (G) Western blot analysis of p21 and p16 in control and SIRT1‐knockdown HCA2‐hTERT cells. (H) Relative mRNA levels of CCL2 and MMP3 in control and SIRT1‐overexpressing HCA2‐hTERT cells. (I) Relative mRNA levels of CCL2 and MMP3 in control and SIRT1‐knockdown HCA2‐hTERT cells. The cells were treated with DMSO or 3TC. H, I: bars represent mean ± SD of biological replicates. Two‐tailed Student's *t*‐test. C: bars represent mean ± SEM Mann–Whitney *U* test. Elements created with Figdraw.com.

During cellular senescence, L1 retrotransposon undergoes transcriptional activation, which subsequently triggers the cGAS‐STING signaling pathway (Cai et al. 2014; Mathavarajah and Dellaire 2024; Gamdzyk et al. 2020; Bregnard et al. 2016). Given that SIRT1 suppresses L1, we hypothesized that SIRT1 might regulate cellular senescence through inhibiting L1 transcription. To test this, we induced cellular senescence in HCA2‐hTERT and HeLa cells using X‐Ray irradiation and assessed senescence markers in control versus SIRT1‐overexpressing or SIRT1‐deficient cells (Figure 2A).

We irradiated the HCA2‐hTERT and HeLa cells with X‐Ray at doses of 10 and 8 Gy, cultured them for 7–10 days and then stained them with SA‐β‐gal. The analysis revealed that SIRT1 overexpression significantly decreased the percentage of SA‐β‐gal‐positive cells (from ~30% to ~13% in HCA2‐hTERT; from ~38% to ~20% in HeLa) (Figure 2B–D, Figure S2A,B), whereas SIRT1 knockdown increased it by 10% (Figure S2C,D). Consistent with this, the expression of the senescence markers p21 and p16 was significantly decreased in SIRT1‐overexpressing cells (Figure 2E, Figure S2E) and increased in SIRT1‐knockdown cells (Figure 2F,G, Figure S2F). We next examined the senescence‐associated secretory phenotype (SASP), a hallmark of senescence defined by the sustained secretion of numerous factors. RT‐qPCR quantification of SASP factors revealed that SIRT1 overexpression significantly downregulated their expression, while SIRT1 knockdown led to their upregulation (Figure 2H,I, Figure S2G,H). L1 can stimulate the cGAS‐STING pathway (Cai et al. 2014; Mathavarajah and Dellaire 2024; Gamdzyk et al. 2020; Bregnard et al. 2016). We found that the phosphorylation levels of TBK1 and STING were decreased in SIRT1‐overexpressing cells and induced to high levels in SIRT1‐knockdown cells (Figure S2I,J).

Several studies have reported that nucleoside reverse transcriptase inhibitors (NRTIs) such as 3TC are potent inhibitors of L1 (Simon et al. 2019; Jones et al. 2008). In order to assess the efficacy of 3TC in inhibiting L1 in the context of SIRT1‐knockdown cells, we examined the markers for senescent cells such as SASP factors (Figure 2I). Together, these results indicate that 3TC attenuates SIRT1 deficiency‐induced cellular senescence.

### 2.3 SIRT1 is Enriched at L1 Loci Under Quiescence

**FIGURE 3:**
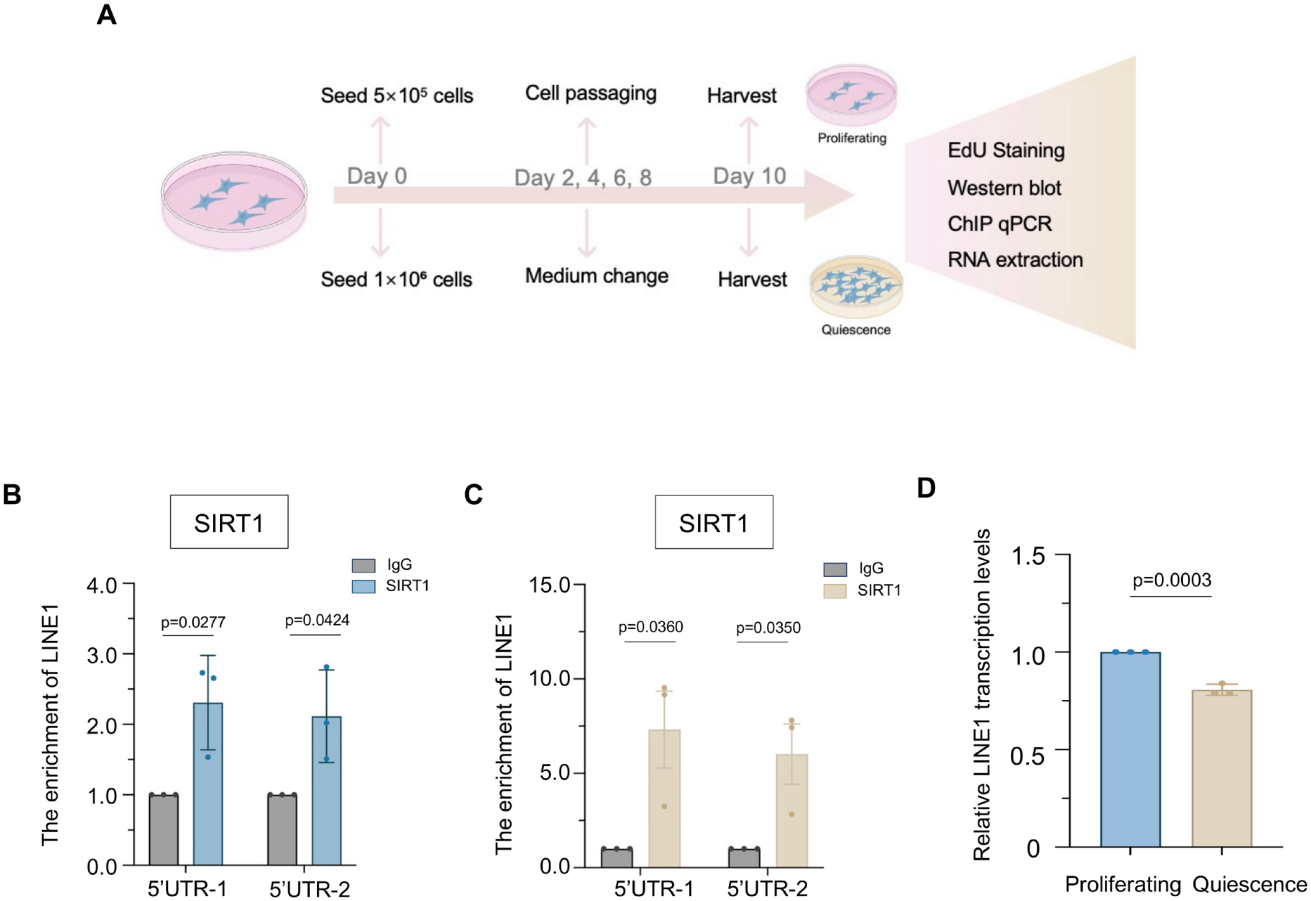
SIRT1 is enriched at L1 loci under quiescence. (A) Schematic diagram illustrating the protocol for establishing proliferating and quiescent cell cultures. (B) ChIP‐qPCR assay showing the enrichment of SIRT1 at indicated regions of L1 in IMR90‐SVLT cells under proliferative conditions. Pre‐immune IgG served as the negative control. (C) ChIP assay showing the enrichment of SIRT1 at indicated regions of L1 in IMR90‐SVLT cells under quiescent conditions. Pre‐immune IgG served as the negative control. (D) Analysis of L1 mRNA levels in IMR90‐SVLT cells under proliferative and quiescent conditions. B‐D: bars represent mean ± SD of biological replicates. Two‐tailed Student's *t*‐test. Elements created with Figdraw.com.

A previous work established that cell division is required for L1 retrotransposition (Shi et al. 2007). We then hypothesized that SIRT1‐mediated suppression of L1 is more potent in quiescent cells. Indeed, the chromatin immunoprecipitation (ChIP) analysis in mouse skeletal muscle satellite cells exhibited a significant increase in SIRT1 enrichment at L1 loci under quiescent conditions (Ryall et al. 2015) (Figure S3A). To validate this finding, we cultured both proliferating cells and quiescent cells induced by contact inhibition upon reaching confluence (Figure 3A). Quiescence was confirmed by markedly reduced EdU incorporation and decreased Ki67 expression, both well‐established markers of quiescent cells (Mitra et al. 2018) (Figure S3B–D). Subsequent ChIP analysis demonstrated that endogenous SIRT1 binds to the 5′‐UTR of L1 and that its enrichment at L1 loci is significantly higher in quiescent cells than in proliferating cells (Figure 3B,C). Consistent with the repressive function of SIRT1 in L1 transcription, we observed a reduction in L1 transcript levels in quiescent cells (Figure 3D). These data suggest that the cell cycle dependence of L1 retrotransposition is due, at least in part, to the transcriptional repression by SIRT1.

### 2.4 Heterochromatin Stabilization Through SIRT1‐Mediated Lamin B1‐KAP1 Cooperativity

**FIGURE 4:**
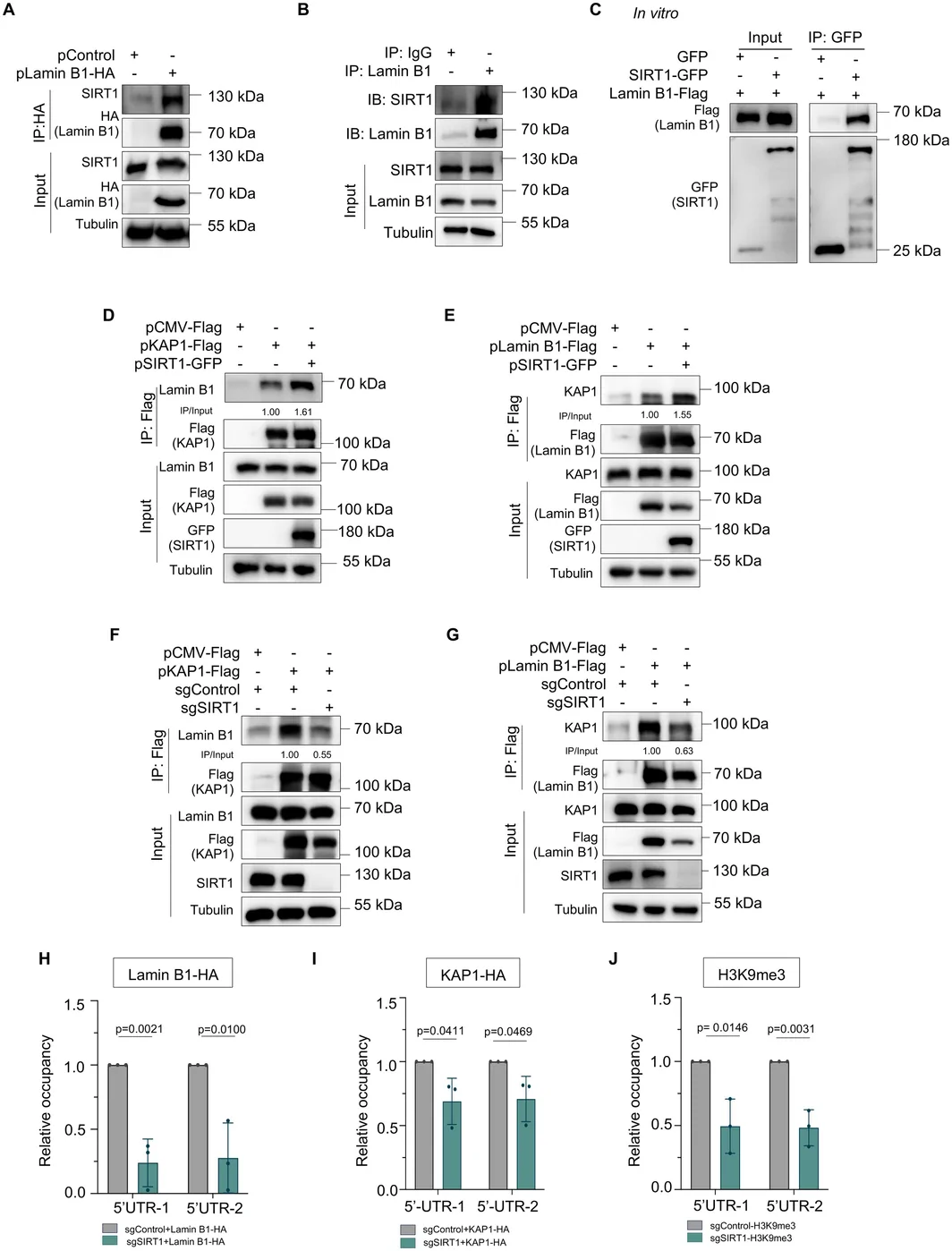
Heterochromatin stabilization through SIRT1‐mediated Lamin B1‐KAP1 cooperativity. (A) Co‐IP analysis showed the interaction between Lamin B1 and SIRT1 in HEK293FT cells. (B) Co‐IP assay of the interaction between Lamin B1 and SIRT1 using antibodies to Lamin B1. (C) Analysis of the interaction between SIRT1 and Lamin B1 in vitro with purified GFP‐tagged SIRT1 and Flag‐tagged Lamin B1 proteins. (D, E) Co‐IP assay of Lamin B1 and KAP1 in HEK293FT cells with or without SIRT1 overexpression, with immunoprecipitation performed against KAP1‐Flag (D) and Lamin B1‐Flag (E), respectively. (F, G) Co‐IP assay of Lamin B1 and KAP1 in control and SIRT1‐knockout HEK293FT cells, with immunoprecipitation performed against KAP1‐Flag (F) and Lamin B1‐Flag (G), respectively (H–J) ChIP assay showing the enrichment of Lamin B1 (H), KAP1 (I) and HeK9me3 (J) at indicated regions of L1 in control and SIRT1‐knockout cells. Pre‐immune IgG served as the negative control. H–J: bars represent mean ± SD of biological replicates. Two‐tailed Student's *t*‐test.

To investigate the regulatory mechanism for enhanced L1 suppression in quiescence, we performed immunoprecipitation (IP) with an anti‐SIRT1 antibody followed by liquid chromatography–tandem mass spectrometry (LC–MS/MS) analysis in IMR90‐SVLT cells under quiescent and proliferative conditions (Figure S4A). Previous studies have demonstrated a link between L1 retrotransposition and heterochromatin state (Della Valle et al. 2022; Chow et al. 2010). This approach identified a set of SIRT1‐interacting proteins implicated in heterochromatin organization, including the nuclear lamin protein Lamin B1 and the heterochromatin protein KAP1 (Figure S4A). Notably, these interactions were specific to quiescent conditions. Reciprocal co‐IP experiments demonstrated that SIRT1 interacted with Lamin B1 in HEK293FT cells (Figure 4A,B). Moreover, in vitro co‐IP experiments with purified recombinant SIRT1 and Lamin B1 proteins confirmed that the two proteins interacted directly (Figure 4C). Physical interaction between SIRT1 and KAP1 has been experimentally validated in prior studies (Lin et al. 2015).

Subsequently, we examined whether SIRT1 could regulate the protein levels of Lamin B1 and KAP1. Western blot analysis revealed that neither overexpression nor knockdown of SIRT1 significantly altered the protein levels of Lamin B1 or KAP1 (Figure S4B,C). Previous studies have indicated that KAP1 interacts with Lamin B1 and that they cooperate to maintain heterochromatin stability (Yang et al. 2024). Surprisingly, we found that SIRT1 overexpression enhances the interaction between Lamin B1 and KAP1, while SIRT1 knockout inhibited this interaction (Figure 4D–G), a finding corroborated by endogenous co‐IP assays (Figure S4D,E).

To investigate whether SIRT1 knockout activates L1 expression and promotes stress‐induced cellular senescence by dissociating heterochromatin maintenance proteins, we performed ChIP experiments. Our data showed that Lamin B1 and KAP1 bound to specific L1 promoter regions in control cells, but this binding was abolished in SIRT1‐knockout cells (Figure 4H,I). Given that constitutive heterochromatin is marked by heterochromatin protein 1α (HP1α), which recognizes and binds to H3K9me3, we assessed this histone mark. Western blot analysis revealed a global decrease in H3K9me3 levels upon SIRT1 knockdown (Figure S4F). Consistently, ChIP assays demonstrated reduced H3K9me3 enrichment at L1 promoters in the absence of SIRT1 (Figure 4J). Collectively, these findings demonstrate that SIRT1 facilitates the recruitment of heterochromatin‐associated factors to L1 elements, thereby maintaining chromatin integrity and suppressing L1 retrotransposition.

## 3 Discussion

SIRT1, the most extensively studied member of the longevity sirtuin family, has been demonstrated to regulate the aging process and the onset of age‐related diseases (Zhao et al. 2020; Wu et al. 2022). However, whether SIRT1 modulates cellular senescence by targeting L1 retrotransposons remains unexplored. In this study, we identified a novel signaling pathway through which SIRT1 regulates cellular senescence. SIRT1 recruits the heterochromatin‐associated factors Lamin B1 and KAP1 to remodel chromatin structure, thereby suppressing L1 retrotransposon activity and ultimately delaying stress‐induced cellular senescence.

SIRT1 participates in the regulation of cellular senescence and organismal aging through its NAD^+^‐dependent deacetylase activity (Canto and Auwerx 2012). It modulates multiple factors involved in senescence by deacetylating targets such as p53, thereby preventing UVB‐induced skin senescence in vivo (Dai et al. 2025). SIRT1 also deacetylates FoxO1 to inhibit oxidative stress and apoptosis, thereby protecting the heart from ischemia–reperfusion (I/R) injury (Hsu et al. 2010). Additionally, SIRT1 promotes DNA double‐strand break repair (DSBR), nucleotide excision repair (NER), and base excision repair (BER) pathways by deacetylating DNA repair proteins such as Ku70, XPA, and APE1, thereby contributing to genomic stability (Jeong et al. 2007; Yamamori et al. 2010; Mei et al. 2016; Kciuk et al. 2020). SIRT1 can also function in a deacetylase‐independent manner (Kang et al. 2011; Campagna et al. 2011). For instance, it stabilizes the tumor suppressor PML both in vitro and in vivo independently of its enzymatic activity (Campagna et al. 2011). In this study, we demonstrate that SIRT1 regulates heterochromatin‐associated proteins and influences L1 retrotransposition via a mechanism that does not depend on its enzymatic activity. Although we confirmed that SIRT1 overexpression rescued the ORF2‐induced increase in genomic instability, its effect on genomic stability may not be entirely attributable to its regulation of L1, given that SIRT1 is also involved in the DNA damage response and DSB repair as mentioned above.

Other Sirtuins, such as SIRT6 and SIRT7, have been reported to influence L1 retrotransposition by modulating heterochromatin factors, suggesting that members of the Sirtuin family share relatively similar functions (Bi et al. 2020; Van Meter et al. 2014). Mechanistically, SIRT6 utilizes its enzymatic activity to ADP‐ribosylate KAP1 and promotes the interaction between KAP1 and the heterochromatin factor HP1α (Van Meter et al. 2014). SIRT7 mediates deacetylation of H3K18, which promotes the association of L1 with lamin proteins (Vazquez et al. 2019). Additionally, its downregulation during hMSC senescence leads to detachment of lamina‐associated domains (LADs) from the nuclear lamina, resulting in loss of heterochromatin and derepression of L1 (Bi et al. 2020). In contrast, SIRT1 suppresses L1 expression by modulating the interaction with chromatin‐associated factors to influence H3K9me3 levels (Adamkova et al. 2017). Although they all regulate heterochromatin state, Sirtuin family members have evolved distinct mechanisms to mediate L1 activity.

It is known that L1 retrotransposition is dependent on the cell cycle (Shi et al. 2007; Xie et al. 2013). Under quiescent conditions, L1 transcription is suppressed. In this study, we propose that during quiescence, enhanced binding of SIRT1 to the L1 5′‐UTR region contributes to the suppression of L1 transcription and retrotransposition. In senescent cells, however, L1 transcription becomes activated despite cell cycle arrest at the G1 phase. Notably, SIRT1 expression is significantly downregulated in both cellular and organismal aging (Xu et al. 2020). We therefore hypothesize that the downregulation of SIRT1 in senescent cells leads to L1 derepression.

Prior studies have established that SUV39H1/2‐dependent H3K9me3 levels regulate L1 expression and that SIRT1 can target SUV39H1 (Vaquero et al. 2007). However, our mass spectrometry data did not reveal enhanced interaction between SIRT1 and SUV39H1 under quiescent conditions. It has been reported that KAP1 recruits the histone methyltransferase SETDB1, heterochromatin protein 1 (HP1), and the NuRD histone deacetylase complex to modulate H3K9me3, thereby influencing endogenous retroelements (Rowe et al. 2010). Furthermore, heterochromatin‐mediated anchoring of transposable elements is dependent on lamin proteins. Here, we demonstrate that SIRT1 suppresses L1 transcription by binding to both KAP1 and Lamin B1, thereby regulating H3K9me3 levels at the L1 5′‐UTR promoter region.

Although our study demonstrates that SIRT1 can delay cellular senescence by suppressing L1 retrotransposon activity, the senescence models examined here are limited to stress‐induced senescence, not replicative senescence. Additionally, most of our mechanistic experiments were not validated in primary cells, which may limit the generalizability of SIRT1‐mediated L1 suppression in delaying aging under physiological conditions.

In conclusion, we have elucidated a novel regulatory pathway through which SIRT1 influences stress‐induced cellular senescence. We demonstrate that SIRT1 suppresses L1 transcription by promoting its interaction with KAP1 and Lamin B1, consequently regulating H3K9me3 levels at the L1 5′‐UTR promoter region. These findings establish a new mechanistic framework for SIRT1‐mediated senescence intervention, with profound implications for delaying aging and mitigating age‐related diseases.

## 4 Materials and Methods

### 4.1 Cell Culture

IMR90‐SVLT cells and HCA2‐hTERT were cultured in DMEM (HyClone, Cat# SH30234) supplemented with 10% fetal bovine serum (Gibco), 1% nonessential amino acids (Gibco) and 1% penicillin–streptomycin (Gibco). HeLa cells and HEK293FT cells were cultured in DMEM (Corning, Cat# 10‐013‐CVR) containing 10% fetal bovine serum (Gibco) and 1% penicillin–streptomycin (Gibco). All cell lines were maintained at 37°C in a humidified atmosphere with 5% CO_2_ (Thermo Fisher HERAcell 240i) and routinely screened for mycoplasma contamination by PCR.

### 4.2 Reagents and Plasmids

The open reading frames (ORFs) of SIRT1, fused with either Flag or GFP at the C‐terminus, were cloned into the pEGFP‐N1 backbone. The ORFs of KAP1 and Lamin B1, fused with HA or Flag at the C‐terminus, were also cloned into the pEGFP‐N1 backbone. The coding sequences (CDSs) of LINE‐1 ORF1p and ORF2p were PCR‐amplified from HeLa cDNA and subsequently cloned into a CMV promoter‐driven vector containing an N‐terminal Flag or HA tag. Mutants of SIRT1 were generated using the wild‐type plasmids as templates. Viral vectors expressing shRNAs were constructed based on the pLKO.1 vector.

The shRNA sequences were as follows:

- shSIRT1‐1, 5′‐CACCGAACAGGTTGCGGGAATCCAA‐3′;
- shSIRT1‐2, 5′‐AAACTTGGATTCCCGCAACCTGTTC‐3′;

The siRNA sequences were as follows:

- siSIRT1(human), 5′‐CAUGAAGUGCCUCAGAUAUUA‐3′;
- siSirt1(mouse), 5′‐GAAGUUGACCUCCUCAUUGU‐3′.

The antibodies employed in the study included anti‐SIRT1 (CST, Cat. #D739); anti‐KAP1 (ABclonal, Cat. #A19568), anti‐Lamin B1 (ABclonal, Cat. #A11495), anti‐p21 (ABclonal, Cat. #A19094), anti‐p16 (ABclonal, Cat. #A23882), anti‐TBK1 (CST, Cat. #3504), anti‐Phospho‐TBK1 (CST, Cat. #5483), anti‐STING (ABclonal, Cat. #A21051), anti‐Phospho‐STING (ABclonal, Cat. #AP1369), anti‐H3K9me3 (CST, Cat. #5326), anti‐Flag (ABclonal, Cat. #AE005), anti‐GFP (Abcam, Cat. #ab290), anti‐HA tag (CST, Cat. #3724), anti‐γH2AX (S139) (CST, Cat. #9718), anti‐Tubulin (ABclonal, Cat. #AC007), anti‐rabbit IgG‐HRP (Bio‐Rad, Cat. #170–6515), anti‐rabbit IgG H&L DyLight 488 (Abcam, Cat. #ab96899) and anti‐mouse IgG H&L DyLight 594 (Abcam, Cat. #ab96881).

### 4.3 L1 Retrotransposition Assay

The retrotransposition efficiency was analyzed according to a previously reported protocol (Zhen et al. 2023). Briefly, HeLa cells were seeded into 6well plates at a density of 2 × 10^4^ cells per well. On Day 2, cells were cotransfected with 2 μg of the pEGFP‐LRE3 reporter plasmid, 1.5 μg of DsRed2, and increasing amounts of SIRT1‐encoding constructs, using the CN114 program on a Lonza 4D Nucleofector system. The culture medium was replaced with fresh medium containing 3 μg/mL puromycin twice, on Day 3 and on Day 8. On Day 10, surviving cells were harvested and analyzed by flow cytometry (BD Biosciences). Retrotransposition efficiency was calculated as the ratio of GFP‐positive cells to DsRed2‐positive cells, and normalized values were reported as normalized relative L1 retrotransposition efficiency. A minimum of 20,000 events were acquired per sample. For samples with notably low retrotransposition frequencies, up to 50,000 events were recorded.

### 4.4 Cellular Quiescence

To induce quiescence, the cells were cultured to confluence. Specifically, the cells were plated at a density of 1 × 10^6^ cells per plate and harvested on Day 10 post‐seeding.

### 4.5 Alkaline Comet Assay

Cells transfected with the indicated plasmids were collected 24 h after transfection and processed for the alkaline comet assay according to the supplier's recommendations (Trevigen, #4250‐050‐K). Genimic instability was evaluated by quantifying tail moments using CometScore software (Cas‐plab v1.2.3b2), with a minimum of 100 cells scored per experimental group.

### 4.6 Luciferase Assay

Cells were co‐transfected with the indicated firefly luciferase reporter plasmids and the pRL‐SV40 *Renilla* control vector. At 24 h post‐transfection, cells were harvested and lysed according to the manufacturer's protocol. Relative luciferase activity was measured using the Dual‐Luciferase Reporter Assay System (Promega, #E1910) on a GloMax Luminometer (Promega, #E5311). All experiments were performed in at least three independent biological replicates.

### 4.7 RNA Extraction and Real‐Time Quantitative PCR (RT‐qPCR)

Total RNA was extracted using the RNA simple Total RNA Kit (TIANGEN) and subsequently reverse transcribed into cDNA using the TransScript II Reverse Transcriptase kit (TRANS). Briefly, RT‐qPCR was performed for L1 and SASP genes using FastStart DNA Master SYBR Green Mix (Roche, Cat. #4913914001) on a ViiA 7 Real‐Time PCR system (Applied Biosystems). The average threshold cycle (Ct) of quadruplicate reactions was determined, and expression was analyzed by the ΔΔCt method. The relative expression levels were normalized to the level of GAPDH. The primers used to amplify were as follows:

- ORF1‐1‐F, 5′‐TCCTCGAGAAGAGCAACTCC‐3′;
- ORF1‐1‐R, 5′‐TGTAGGGTTTCTGCCGAGAG‐3′;
- ORF1‐2‐F, 5′‐GGCCAACGTTCAGATTCAGG‐3′;
- ORF1‐2‐R, 5′‐CTTTGAGGGTAACCCGACCT‐3′;
- ORF2‐1‐F, 5′‐CGAATCCAGCAGCACATCAA‐3′;
- ORF2‐1‐R, 5′‐ATTGAACCAGCCTTGCATCC‐3′;
- ORF2‐2‐F, 5′‐GGCGAAGGACATGAACAGAC‐3′;
- ORF2‐2‐R, 5′‐TTTCTCCGCATCCTCTCCAG‐3′;
- CCL2‐F, 5′‐AGTTCTTGCCGCCCTTCT‐3′;
- CCL2‐R, 5′‐GTGACTGGGGCATTGATTG‐3′;
- MMP3‐F, 5′‐GCAGTTTGCTCAGCCTATCC‐3′;
- MMP3‐R, 5′‐GAGTGTCGGAGTCCAGCTTC‐3′;
- IL‐1α‐F, 5′‐GGTTGAGTTTAAGCCAATCCA‐3′;
- IL‐1α‐R, 5′‐TGCTGACCTAGGCTTGATGA‐3′;
- IL‐1𝛽‐F, 5′‐CTGTCCTGCGTGTTGAAAGA‐3′;
- IL‐1𝛽‐R, 5′‐TTGGGTAATTTTTGGGATCTACA‐3′;
- IL‐6‐F, 5′‐GCCCAGCTATGAACTCCTTCT‐3′;
- IL‐6‐R, 5′‐GAAGGCAGCAGGCAACAC‐3′;
- mL1‐1‐F, 5′‐TAAGAGAGCTTGCCAGCAGAGA‐3′;
- mL1‐1‐R, 5′‐GCAGACCTGGGAGACAGATTCT‐3′;
- mL1‐2‐F, 5′‐TGGAAGAGAGAATCTCAGGTGC‐3′;
- mL1‐2‐R, 5′‐TTGTGCCGATGTTCTCTATGG‐3′;
- GAPDH‐F, 5′‐ATGACATCAAGAAGGTGGTG‐3′;
- GAPDH‐R, 5′‐CATACCAGGAAATGAGCTTG‐3′.

### 4.8 Co‐Immunoprecipitation (Co‐IP)

Cells transfected with the indicated plasmids were harvested 24 h post‐transfection and lysed for 30 min on ice in lysis buffer (20 mM HEPES (pH 8.0), 150 mM NaCl, 0.2 mM EDTA, 1% NP‐40 and 5% glycerol) supplemented with protease inhibitor cocktail. The lysates were then sonicated at 10% amplitude for a total of 2 min with 2 s on/off pulses, followed by centrifugation at 13,500 rpm for 15 min at 4°C. The supernatants were incubated with antibody‐conjugated beads overnight at 4°C. Beads were subsequently washed four times with ice‐cold lysis buffer, resuspended in 2 × sample buffer, and boiled for 10 min prior to Western blot analysis.

### 4.9 Chromatin Immunoprecipitation (ChIP) Assay

ChIP assays for the enrichment of H3K9me3, Lamin B1 and KAP1 at L1 genomic regions were performed as described previously (Chen et al. 2019; Liu et al. 2018). Cells were cultured for 24 h and then harvested for the ChIP assay. Following immunoprecipitation, the chromatin and input fragments were used as templates for qPCR with the following primers:

- 5′‐UTR‐1‐F, 5′‐ACGAGACTATATCCCACACCT‐3′;
- 5′‐UTR‐1‐R, 5′‐GCAGAGGTTACTGCTGTCTT‐3′;
- 5′‐UTR‐2‐F, 5′‐TGTCTGACAGCTTTGAAGAGAG‐3′;
- 5′‐UTR‐2‐R, 5′‐TGGTCTTTGATGATGGTGATGTA‐3′.

### 4.10 Senescence‐Associated β‐Galactosidase (SA‐β‐Gal) Staining

Cellular senescence was induced by exposure to the indicated doses of ionizing radiation. Seven to 10 days after irradiation, cells were fixed for 5 min at room temperature in PBS containing 2% formaldehyde and 0.2% glutaraldehyde, washed twice with PBS, and then incubated with staining solution (1 mg/mL X‐gal in dimethylformamide, 40 mM citric acid/sodium phosphate buffer, 5 mM potassium ferrocyanide, 5 mM potassium ferricyanide, 150 mM sodium chloride, and 2 mM magnesium chloride). After an overnight incubation at 37°C, bright‐field images of the stained cells were captured.

### 4.11 EdU Incorporation Assay

IMR90‐SVLT cells maintained under proliferating or quiescent conditions were incubated with EdU (5‐ethynyl‐2′‐deoxyuridine) for 2 h. EdU incorporation was then detected using the EdU Cell Proliferation Kit (Sangon Biotech, Cat. #E607204), and images were acquired with a fluorescence microscope equipped with DAPI and red fluorescence filter sets.

### 4.12 LC–MS/MS Analysis

Protein eluates from IP were separated by 10% SDS‐PAGE gel. The gel was stained with Coomassie brilliant blue and then washed with water for several times to destain. After determining molecular weight, bands were excised from the gel for aimed proteins. Samples then underwent dehydration (100% acetonitrile), reduction (10 mM DTT in 25 mmol/L NH_4_HCO_3_ for 45 min at 56°C), and alkylation (40 mmol/L iodoacetamide in 25 mmol/L NH_4_HCO_3_ for 45 min at room temperature in the dark). After sequence‐grade modified trypsin (40 ng for each band) in 25 mmol/L NH_4_HCO_3_, gel bands were dried and digested. Finally, the enzymatic reaction was stopped with formic acid. The resultant solution was identified using nanoLC‐Q Exactive (Thermo Scientific) equipped with data‐dependent mode that allows MS data acquirement at a high resolution of 70,000 (m/z 200) across the mass range of 300–1600 m/z. Using Sequest HT and search engine, all raw files were processed from Q Exactive and analyzed with Proteome Discovery version 1.4 for protein identification and Percolator for FDR (false discovery rate). Our data were analyzed against a Uniprot human protein database (updated on 06‐2013). Percolator used FDR analysis. We defined FDR < 1% for protein identification. The confidence of peptides was set as high for peptides filter. Gene Ontology enrichment analysis was conducted by ToppGene.

### 4.13 Generation of SIRT1‐Knockout Cells Using the CRISPR–Cas9 System

Oligonucleotides targeting human SIRT1 were synthesized, annealed, and ligated into CRISPR‐Cas9 vector PX458. The resulting constructs were then transfected into HeLa cells. GFP‐positive cells were sorted 24 h post‐transfection using a BD FACS AriaII cell sorter. Successful targeting was confirmed by Western blotting with anti‐SIRT1 antibodies and by Sanger sequencing of PCR products amplified from the genomic regions flanking the sgRNA target sites. The indicated oligonucleotide sequences were as follows:

sgSIRT1, 5′‐AACAGGTTGCGGGAATCCAA‐3′.

### 4.14 ChIP–Seq Analysis

Raw ChIP–seq and matched input FASTQ files were obtained from NCBI GEO (GSE64379). Reads were aligned to the mouse reference (GRCm38) with Bowtie2 (‐‐very‐sensitive, allowing up to 10 multimaps per read), and PCR duplicates were removed with sambamba (markdup‐r). RPGC‐normalized coverage bigWigs were generated using deepTools bamCoverage, and log2(ChIP/Input) tracks were derived with bigwigCompare. A total of 2811 full‐length, putatively retrotransposition‐competent (intact; ORF1‐ and ORF2‐intact) L1 loci were obtained from L1Base2. Therefore, this analysis focuses on a functional subset of intact L1 loci rather than all annotated L1 fragments genome‐wide, which are dominated by older/truncated/inactive copies. Visualization was performed with computeMatrix scale‐regions followed by plotProfile.

## Author Contributions

Anke Geng, and Ying Jiang conceived this project and designed the study. Xiaona Wang, Anke Geng and Tianbo Li performed and interpreted the experiments. Meng xiao Liu, Xiao Huang, Qinghua Peng and Huanyin Tang performed experiments. Anke Geng and Xiaona Wang wrote the manuscript. Zhiyong Mao helped with the proofreading of the manuscript. All the authors reviewed and approved the manuscript.

## Funding

This work was supported by the National Natural Science Foundation of China (32470796 and 32200595).

## Conflicts of Interest

The authors declare no conflicts of interest.

## Supporting information


**Figure S1:** Related to Figure1. (A) Schematic diagram showing the L1 retrotransposition efficiency reporter pEGFP‐LRE3. The reporter construct consists of a full‐length L1 element (containing 5′ and 3′ UTRs and two ORFs). An EGFP retrotransposition cassette is inserted in the antisense orientation into the 3′‐UTR. This cassette comprises a CMV immediate‐early promoter (pCMV) driving expression of an EGFP gene that is disrupted by a sense‐orientation γ‐globin intron (with splice donor, SD, and splice acceptor, SA, sites indicated), followed by a TK poly(A) signal (pA). (B) Western blot analysis of SIRT1 expression in HeLa cells. (C) Representative images of FACS. (D) Western blot analysis of SIRT1 in control and SIRT1‐knockdown HeLa cells. (E) Effects on L1 retrotransposition efficiency in control and SIRT1‐knockdown HeLa cells. (F) Representative images of FACS. (G) Western blot analysis of SIRT1 expression in control and SIRT1‐deficient HeLa cells. (H) Western blot analysis of SIRT1 expression in control and SIRT1‐knockdown IMR90 cells. (I) Analysis of L1 mRNA levels in control and SIRT1‐deficient IMR90 cells. (J) Western blot analysis of SIRT1 expression in control and SIRT1‐knockdown MEFs. (K) Analysis of L1 mRNA levels in control and SIRT1‐deficient MEFs (mL1‐1 corresponds to the m5′ UTR, and mL1‐2 corresponds to mORF1). (L) Analysis of the firefly luciferase gene driven by 5′‐UTR in SIRT1 overexpression IMR90‐SVLT cells. (M) Analysis of the firefly luciferase gene driven by 5′‐UTR in SIRT1‐knockdown IMR90‐SVLT cells. (N) The genomic instability analysis in control and SIRT1‐depleted HeLa cells was performed using the comet assay, with ORF2 overexpressed in each group. The tail moment was employed as the measure of genomic instability, and at least 100 cells were analyzed using the software CometScore. Representative images of the comet assay. (O) The genomic instability analysis was performed in control and in SIRT1 overexpressing IMR90‐SVLT cells using the comet assay, with ORF2 overexpressed in each group. The tail moment was employed as the measure of genomic instability, and at least 100 cells were analyzed using the software CometScore. Representative images of the comet assay. (P) The genomic instability analysis was performed in control and in SIRT1‐knockdown IMR90‐SVLT cells using the comet assay, with ORF2 overexpressed in each group. The tail moment was employed as the measure of genomic instability, and at least 100 cells were analyzed using the software CometScore. Representative images of the comet assay. (Q) Western blot analysis of γH2AX was performed in control and SIRT1‐knockdown HeLa cells, with ORF2 overexpressed in each group. E, I, K, M: bars represent mean ± SD of biological replicates. Two‐tailed Student's *t*‐test. N, O, P: bars represent mean SEM Mann–Whitney *U* test.

**Figure S2:** Related to Figure 2. (A) Quantification of SA‐β‐gal positive HeLa cells. HeLa cells were exposed to 8 Gy of X‐ray irradiation and transfected with the control vector or the SIRT1 overexpression vector for 7 days. Images from different areas of the plate were taken and quantified. (B) Representative images of SA‐β‐gal staining in HeLa cells, which were transfected with the control vector or the SIRT1 overexpression vector (Scale bar: 20 μm). (C) Quantification of SA‐β‐gal positive cells in control and SIRT1‐depleted HeLa cells. Images from different areas of the plate were taken and quantified. (D) Representative images of SA‐β‐gal positive cells in control and SIRT1‐depleted HeLa cells (Scale bar: 50 μm). (E) Western blot analysis of p21 and p16 in control and SIRT1‐overexpressing HeLa cells. (F) Western blot analysis of p21 and p16 in control and SIRT1‐knockdown HeLa cells. (G) Relative mRNA levels of IL‐1α, IL‐1β and IL‐6 in control and SIRT1‐overexpressing HeLa cells. (H) Relative mRNA levels of IL‐1α, IL‐1β and IL‐6 in control and SIRT1‐depleted HeLa cells. (I) Western blot analysis of TBK1 or phosphorylated TBK1 in HeLa cells. (J) Western blot analysis of phosphorylated TBK1 and STING in control and SIRT1‐depleted HeLa cells. G, H: bars represent mean ± SD of biological replicates. Two‐tailed Student's *t*‐test. A, C: bars represent mean SEM Mann–Whitney *U* test. All experiments were repeated three times with similar results.

**Figure S3:** Related to Figure 3. (A) Log2(ChIP/Input) of SIRT1 ChIP–seq profiles across full‐length intact mouse L1 elements, comparing quiescent and proliferating conditions. A total of 2811 full‐length, putatively retrotransposition‐competent (intact; ORF1‐ and ORF2‐intact) L1 loci were obtained from L1Base2. (B) Representative images of EdU‐positive cells in proliferating and quiescent IMR90‐SVLT cells (Scale bar, 50 μm). (C) Quantification of EdU‐positive cells in proliferating and quiescent IMR90‐SVLT cells. Images from different areas of the plate were taken and quantified. (D) Western blot analysis of Ki67 in proliferating and quiescent IMR90‐SVLT cells. C, Data are mean ± SEM Mann–Whitney *U* test.

**Figure S4:** Related to Figure 4. (A) Schematic of mass spectrometry work flow for identifying SIRT1‐interacting proteins in IMR90‐SVLT cells under proliferating and quiescent conditions. Pre‐immune IgG served as the negative control. (B) Western blot analysis of Lamin B1 and KAP1 in HeLa cells. The HeLa cells were transfected with the control vector or the SIRT1 overexpression vector. (C) Western blot analysis of Lamin B1 and KAP1 in control and SIRT1‐deficiency HeLa cells. (D) Co‐IP assay of the interaction between Lamin B1 and KAP1 using antibodies to KAP1 in HEK293FT cells, with SIRT1 overexpression in the last group. (E) Co‐IP assay of the interaction between Lamin B1 and KAP1 using antibodies to KAP1 in control and SIRT1‐deficiency HEK293FT cells. (F) Western blot analysis of H3K9me3 in control and SIRT1‐knockdown HeLa cells, with H3 used as loading control.

## Data Availability

The data that supports the findings of this study are available in the Figures S1–, S4 of this article.
