# Supplementary material for: SIRT1 Silences L1 Retrotransposons by Stabilizing Heterochromatin‐Modifying Complexes
Source: Aging Cell. 2026 Aug 30;25(9):e70689. doi: 10.1111/acel.70689 (PMC13526325; doi:10.1111/acel.70689)
Supplement: Supplementary file 1 [file ACEL-25-e70689-s001.pdf]

A

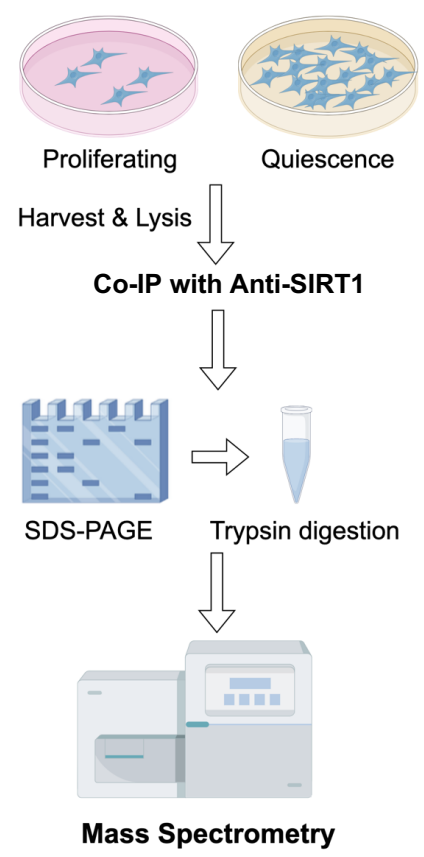

SIRT1 Co-IP MS

Protein	Proliferating	Quiescence
Lamin B1	unfound	474163704
KAP1	unfound	28000854

B

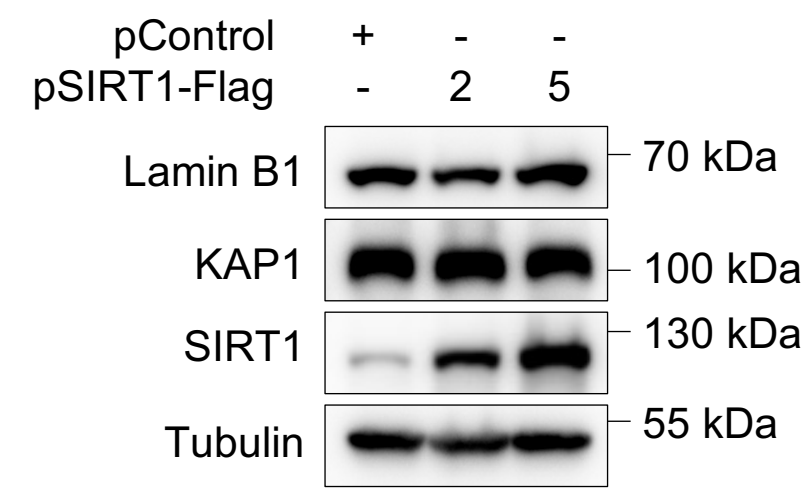

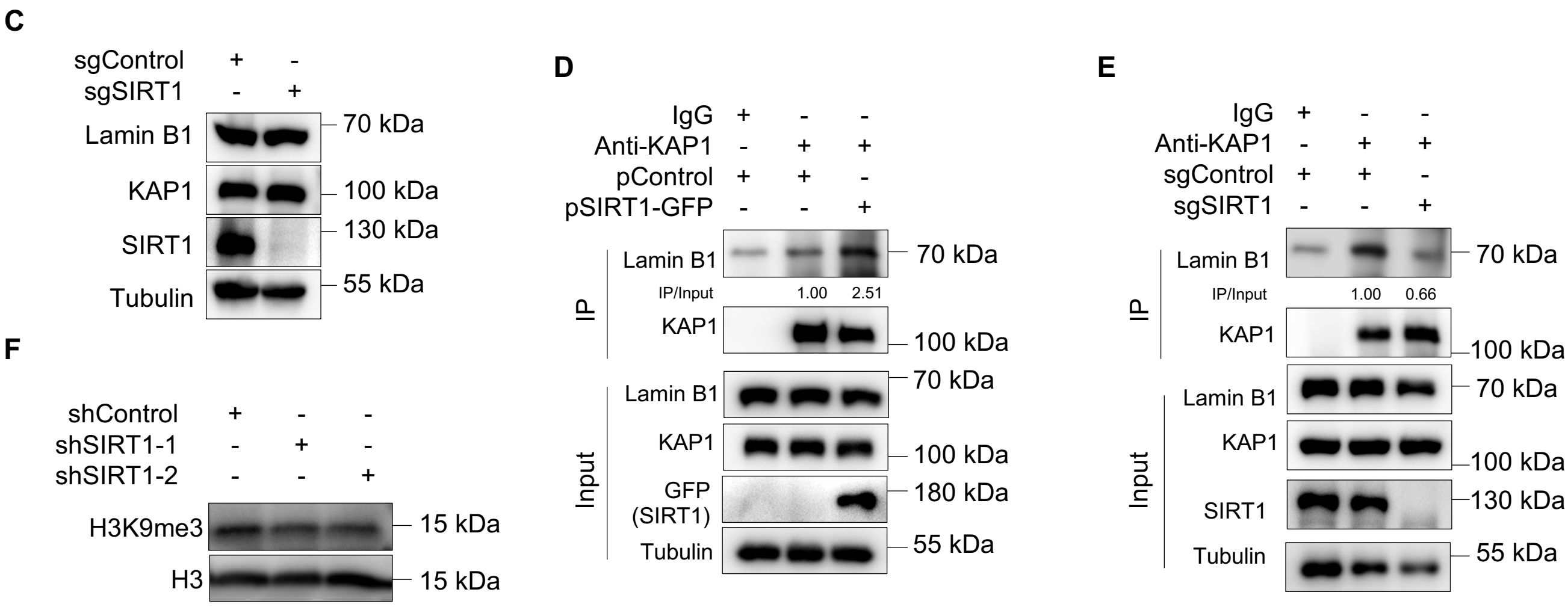

Figure 4s
