# Supplementary material for: SIRT1 Silences L1 Retrotransposons by Stabilizing Heterochromatin‐Modifying Complexes
Source: Aging Cell. 2026 Aug 30;25(9):e70689. doi: 10.1111/acel.70689 (PMC13526325; doi:10.1111/acel.70689)
Supplement: Supplementary file 2 [file ACEL-25-e70689-s002.pdf]

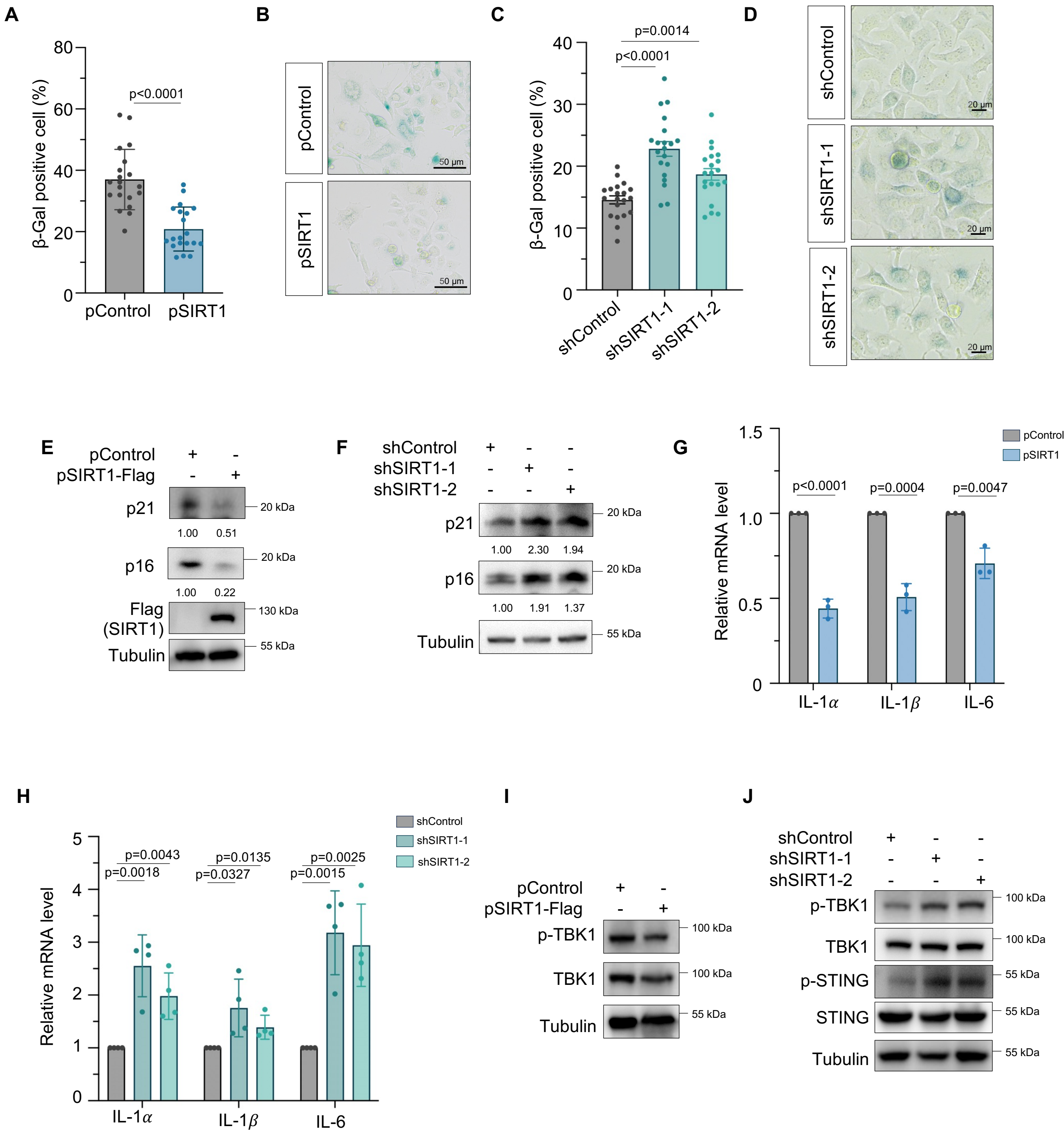

A
B
C
D
β-Gal positive cell (%)
p<0.0001
pControl
pSIRT1
50 μm
p=0.0014
p<0.0001
shControl
shSIRT1-1
shSIRT1-2
20 μm
E
pControl + -
pSIRT1-Flag - +
p21
20 kDa
1.00 0.51
p16
1.00 0.22
Flag (SIRT1)
130 kDa
Tubulin
55 kDa
F
shControl + - -
shSIRT1-1 - + -
shSIRT1-2 - - +
1.00 2.30 1.94
1.00 1.91 1.37
G
Relative mRNA level
p<0.0001
p=0.0004
p=0.0047
IL-1α
IL-1β
IL-6
H
p=0.0043
p=0.0018
p=0.0135
p=0.0327
p=0.0025
p=0.0015
I
p-TBK1
TBK1
100 kDa
J
p-STING
STING

Figure 2s
