# Supplementary material for: SIRT1 Silences L1 Retrotransposons by Stabilizing Heterochromatin‐Modifying Complexes
Source: Aging Cell. 2026 Aug 30;25(9):e70689. doi: 10.1111/acel.70689 (PMC13526325; doi:10.1111/acel.70689)
Supplement: Supplementary file 3 [file ACEL-25-e70689-s003.pdf]

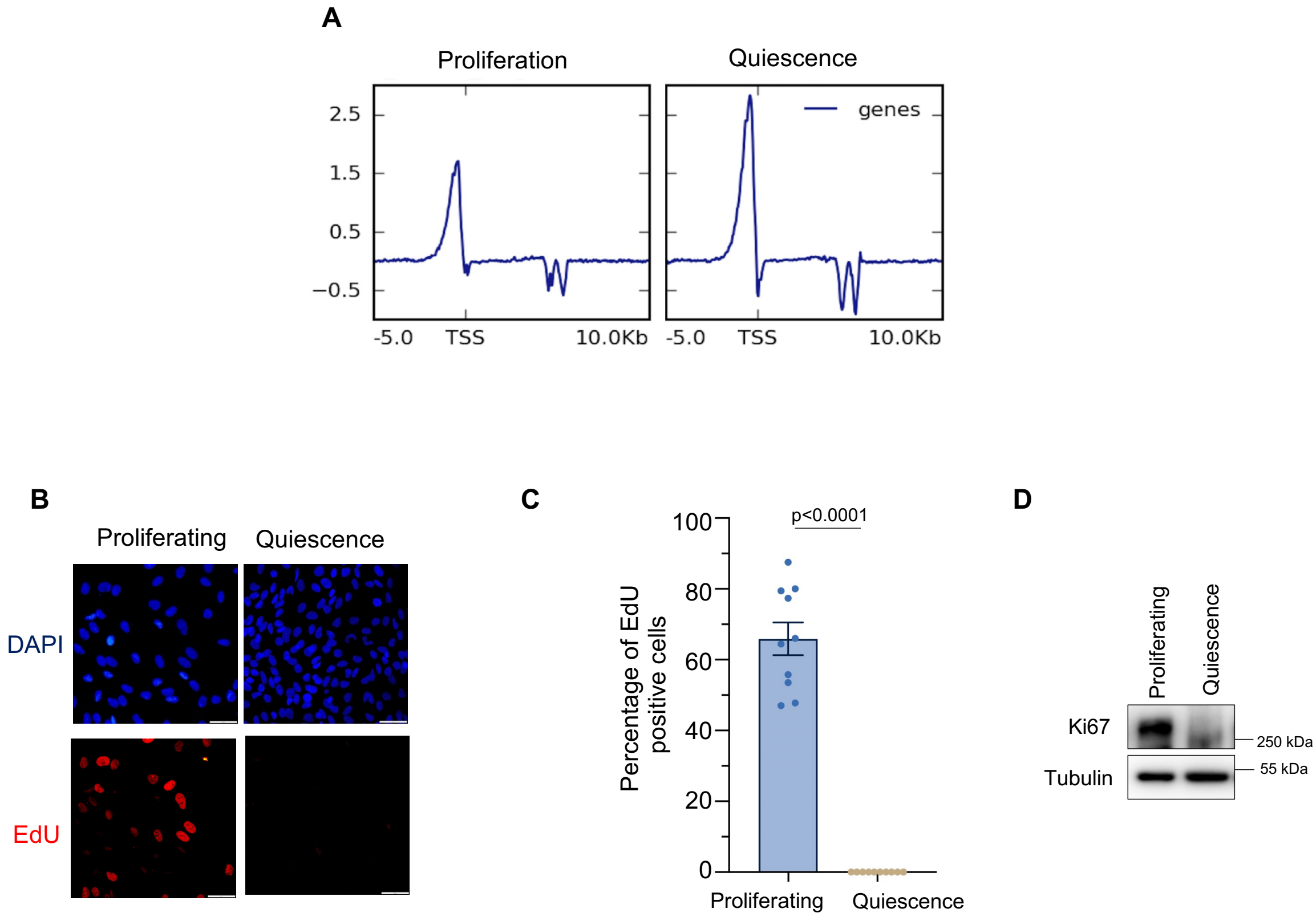

A
Proliferation
Quiescence
genes
2.5
1.5
0.5
−0.5
-5.0
TSS
10.0Kb
B
Proliferating
Quiescence
DAPI
EdU
C
p<0.0001
Percentage of EdU positive cells
100
80
60
40
20
0
Proliferating
Quiescence
D
Proliferating
Quiescence
Ki67
250 kDa
Tubulin
55 kDa

Figure 3s
