# Supplementary material for: SIRT1 Silences L1 Retrotransposons by Stabilizing Heterochromatin‐Modifying Complexes
Source: Aging Cell. 2026 Aug 30;25(9):e70689. doi: 10.1111/acel.70689 (PMC13526325; doi:10.1111/acel.70689)
Supplement: Supplementary file 4 [file ACEL-25-e70689-s004.pdf]

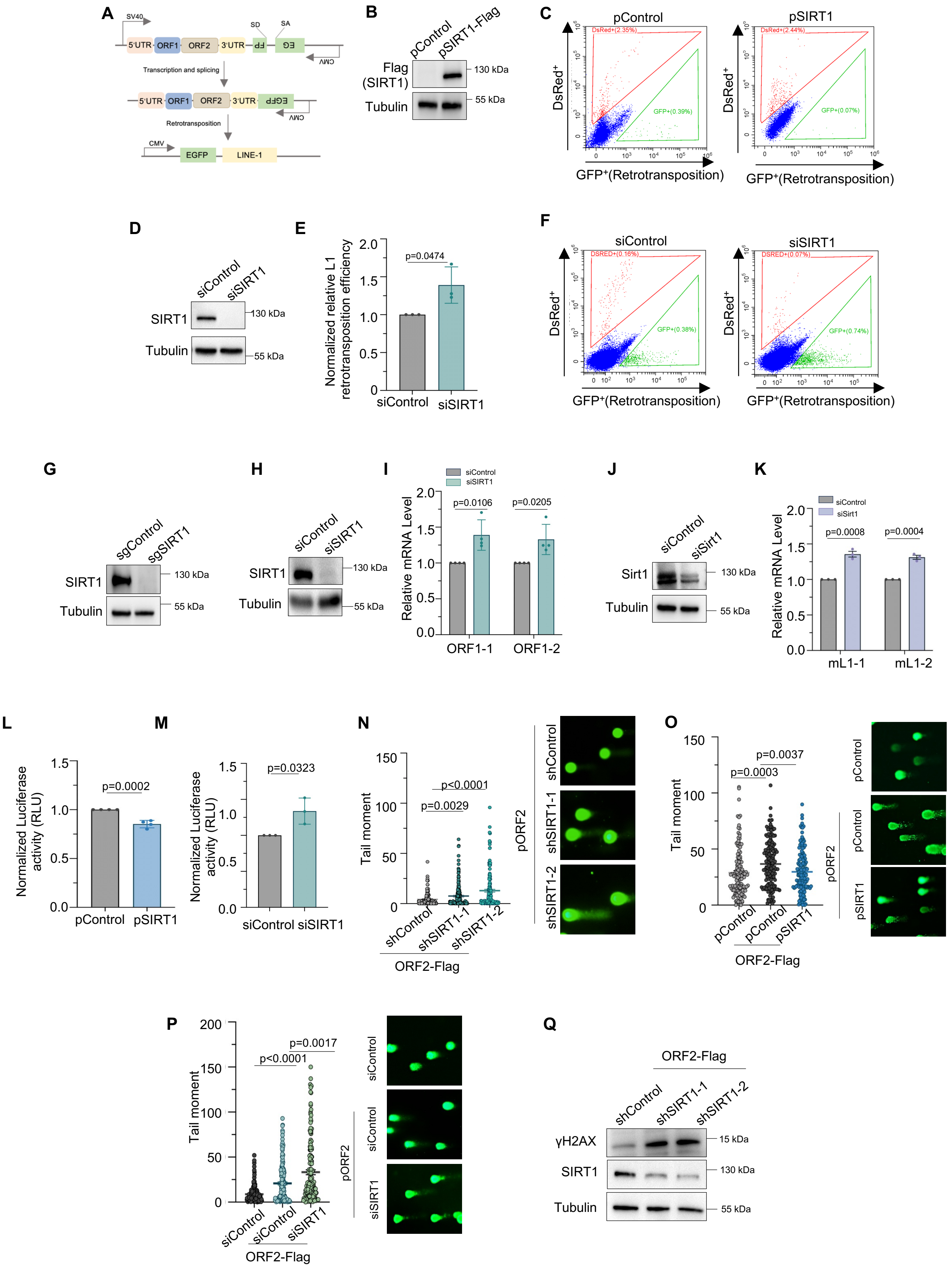
A
SV40
5'UTR ORF1 ORF2 3'UTR
SD
SA
CMV
Transcription and splicing
5'UTR ORF1 ORF2 3'UTR
Retrotransposition
CMV
EGFP
LINE-1
B
pControl
pSIRT1-Flag
Flag (SIRT1)
Tubulin
130 kDa
55 kDa
C
pControl
pSIRT1
DsRed+(2.35%)
DsRed+(2.44%)
GFP+(0.39%)
GFP+(0.07%)
DsRed+
GFP+(Retrotransposition)
D
siControl
siSIRT1
SIRT1
Tubulin
130 kDa
55 kDa
E
p=0.0474
Normalized relative L1 retrotransposition efficiency
siControl
siSIRT1
F
siControl
siSIRT1
DSRED+(0.16%)
DSRED+(0.07%)
GFP+(0.38%)
GFP+(0.74%)
DsRed+
GFP+(Retrotransposition)
G
sgControl
sgSIRT1
SIRT1
Tubulin
130 kDa
55 kDa
H
siControl
siSIRT1
SIRT1
Tubulin
130 kDa
55 kDa
I
siControl
siSIRT1
p=0.0106
p=0.0205
Relative mRNA Level
ORF1-1
ORF1-2
J
siControl
siSirt1
Sirt1
Tubulin
130 kDa
55 kDa
K
siControl
siSirt1
p=0.0008
p=0.0004
Relative mRNA Level
mL1-1
mL1-2
L
p=0.0002
Normalized Luciferase activity (RLU)
pControl
pSIRT1
M
p=0.0323
Normalized Luciferase activity (RLU)
siControl
siSIRT1
N
p<0.0001
p=0.0029
Tail moment
shControl
shSIRT1-1
shSIRT1-2
ORF2-Flag
pORF2
shControl
shSIRT1-1
shSIRT1-2
O
p=0.0037
p=0.0003
Tail moment
pControl
pControl
pSIRT1
ORF2-Flag
pControl
pORF2
pControl
pSIRT1
P
p=0.0017
p<0.0001
Tail moment
siControl
siControl
siSIRT1
ORF2-Flag
siControl
pORF2
siControl
siSIRT1
Q
ORF2-Flag
shControl
shSIRT1-1
shSIRT1-2
γH2AX
SIRT1
Tubulin
15 kDa
130 kDa
55 kDa

Figure 1s
